# Herpesviruses and Inflammasomes: One Sensor Does Not Fit All

**DOI:** 10.1128/mbio.01737-21

**Published:** 2022-01-18

**Authors:** Ayush Kumar, Georgia Stavrakis, Andrew H. Karaba

**Affiliations:** a Ontario Veterinary College, University of Guelph, Guelph, Ontario, Canada; b Department of Medicine, Johns Hopkins University School of Medicine, Baltimore, Maryland, USA; c W. Harry Feinstone Department of Molecular Microbiology and Immunology, Johns Hopkins University Bloomberg School of Public Health, Baltimore, Maryland, USA; Pennsylvania State University; Albert Einstein College of Medicine

**Keywords:** inflammasomes, herpesviruses, innate immunity

## Abstract

Herpesviruses are ubiquitous double-stranded DNA viruses that cause lifelong infections and are associated with a variety of diseases. While they have evolved multiple mechanisms to evade the immune system, they are all recognized by the innate immune system, which can lead to both localized and systemic inflammation. A more recently appreciated mechanism of herpesvirus innate immune activation is through inflammasome signaling. The inflammasome is an intracellular multiprotein complex that, when activated, leads to the release of proinflammatory cytokines, including IL-1β and IL-18, and activation of the inflammatory programed cell death pathway known as pyroptosis. Despite the herpesviruses sharing a similar structure, their mechanisms of inflammasome activation and the consequences of inflammasome activation in cases of virus-associated disease are not uniform. This review will highlight the similarities and differences among herpesviruses with regard to their mechanisms of inflammasome activation and impacts on diseases caused by herpesviruses. Furthermore, it will identify areas where additional studies are warranted to better understand the impact of this important innate immune signaling program on the pathogenesis of these common viruses.

## INTRODUCTION

## OVERVIEW OF INFLAMMASOMES

The discovery of inflammasomes as innate immune signaling complexes has transformed our understanding of the innate immune system. Inflammasomes are intracellular multiprotein complexes that form in response to pathogen recognition and/or danger signals. Classical or canonical inflammasomes are composed of three parts: a sensor protein, a multimeric complex of adaptor-apoptosis-associated-speck-like-protein-containing-a-caspase-recruitment domain (ASC), and caspase-1 (1). The formation of this complex leads to caspase-dependent cleavage of the immature (pro-) forms of the proinflammatory cytokines interleukin (IL)-1β and IL-18, and to cleavage and activation of gasdermin D (GSDMD) (2–4). IL-18 and IL-1β are predominantly produced by myeloid cells, such as macrophages and dendritic cells, and mediate immune responses against pathogens and tissue damage (5). IL-1β induction is a vital initial host defense mechanism during viral and bacterial infections (6). IL-18 is structurally similar to IL-1β and its main functions are mediated through the induction of interferon (IFN)-γ secretion from Th1 cells. Together with IL-12, IL-18 leads to Th1 differentiation that activates both adaptive and innate host defense against intracellular bacteria, viruses, and fungi (5, 7, 8).

**FIG. 1:**
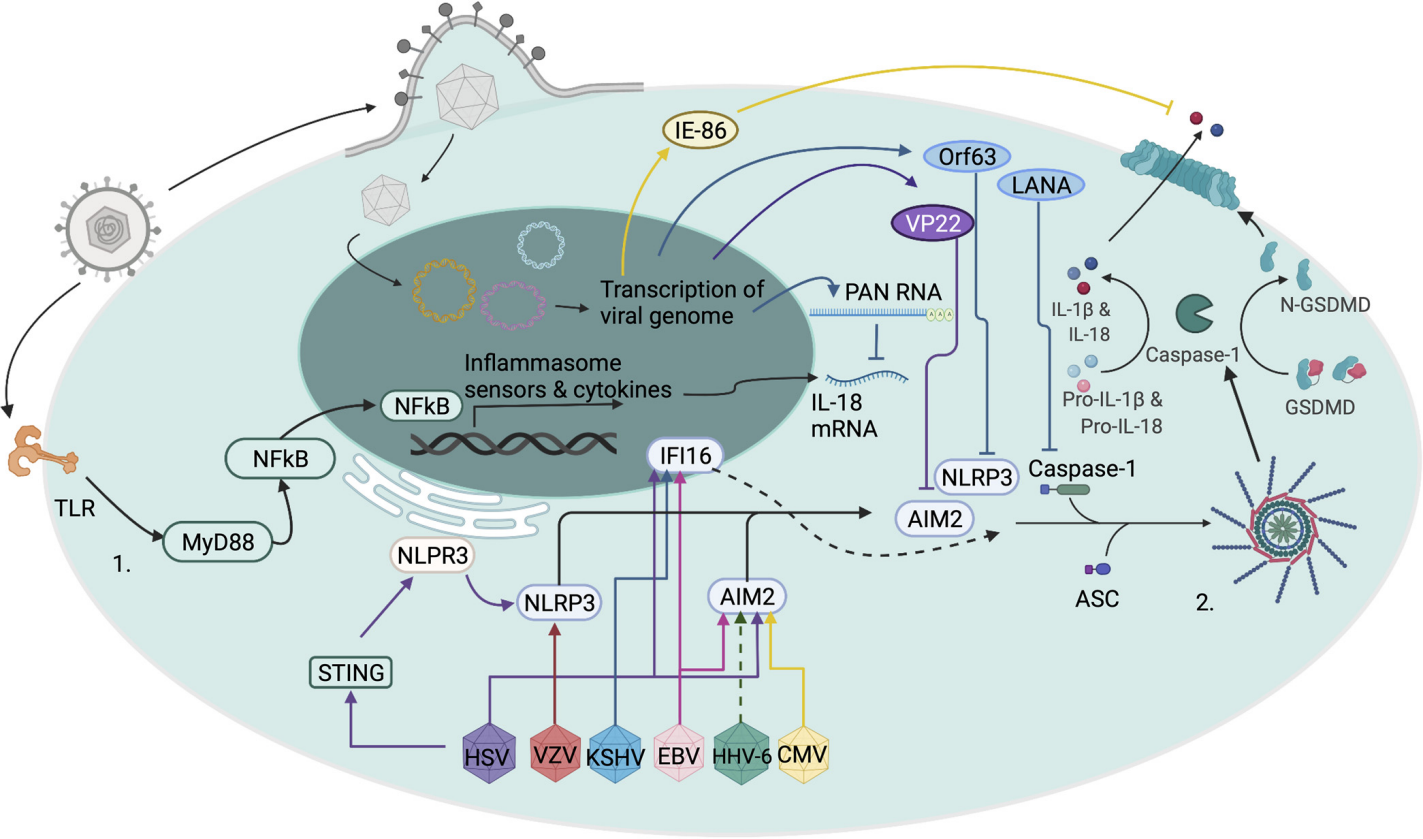
Mechanisms of inflammasome activation and regulation by human herpesviruses. The priming signal (step 1) for inflammasome activation is mediated by Toll-like receptors (TLRs) or other pattern recognition receptors. Activation of TLRs by their microbial ligand leads to downstream signaling via MyD88 to NF-κB. NF-κB enters the nucleus as a transcription factor and increases the expression of inflammasome associated genes (9). The activation signal (step 2) for inflammasome activation during herpesvirus infection results from the sensing of different herpesviruses by cytosolic sensors, such as NLRP3 and AIM2. HSV-1/HSV-2 (HSV) can be sensed by NLRP3 and AIM2 in the cytoplasm and by IFI16 in the nucleus (15, 74, 75). However, the HSV-1 protein VP22 inhibits sensing and inflammasome activation by AIM2 (73). Furthermore, NLRP3 inflammasome activation in response to HSV-1 is mediated via STING (76). VZV activates the inflammasome via NLRP3, whereas CMV activates the inflammasome through AIM2 (17, 54). Additionally, the CMV immediate early 86-KD protein (IE-86) inhibits IL-1β release from infected cells (17). It is hypothesized that HHV-6 activates the inflammasome through AIM2, similarly to CMV. EBV infection can lead to inflammasome activation through AIM2 and IFI16 (84–87). KSHV activation of the inflammasome is initiated via IFI16 sensing in the nucleus which then relocates to the cytoplasm (71, 87, 89). The inflammasome response to KSHV can be blunted by the inhibitory activities of KSHV polyadenylated nuclear RNA (PAN RNA), KSHV Orf63 protein, and LANA (90–92). When these sensors receive the activation signal, they oligomerize with ASC and caspase-1 to form the inflammasome and activate caspase-1 (10). Activated caspase-1 cleaves the pro-forms of inflammasome cytokines, IL-18 and IL-1-β, as well as gasdermin-D (GSDMD) (2–4). Cleaved GSDMD forms a pore at the cell surface which allows for the release of IL-18, IL-1β and the influx of ions leading to pyroptotic cell death (22). Figure created with BioRender.com.

The formation and activation of some of the canonical inflammasomes can be thought of as a two-step process. The first step, priming, requires activation of the nuclear factor κB (NF-κB) pathway. This is often achieved through the detection of pathogen-associated molecular patterns (PAMPs) by pattern recognition receptors (PRRs) such as Toll-like receptors (TLRs). Translocation of NF-κB into the nucleus leads to the transcription of genes critical to inflammasome signaling, including pro-IL-1β, pro-IL-18, and pro-caspase-1 (9). Notably, several inflammasomes do not necessarily require this first step for activation (10, 11). The second step, activation, requires the sensor protein recognizing its cognate signal. This leads to oligomerization of ASC, assembly of the inflammasome, and caspase-1 cleavage of pro-IL-1β and pro-IL-18 (reference 12, Fig. 1). This activation step can be initiated by multiple sensor proteins that can sense a variety of PAMPs and danger-associated molecular patterns (DAMPs). These sensor proteins are often members of the NOD-like-receptor (NLR) family of proteins, such as NLRP1, NLRC4, and NLPR3, and can respond to a diverse array of stimuli. Absent-in-melanoma-2 (AIM2) is another inflammasome sensor that recognizes and forms inflammasomes in response to dsDNA (13, 14). Specific inflammasomes are thus named by their sensor and different sensors are required for inflammasome activation by different viruses. For example, herpes simplex virus 1 (*Human alphaherpesvirus 1*, HSV-1/HHV-1) activates the NLRP3 inflammasome (15–17), while cytomegalovirus *Human betaherpesvirus 5* (CMV/HHV-5) activates the AIM2 inflammasome (18). After recognition of a cognate PAMP or DAMP, these sensor proteins recruit ASC molecules that undergo oligomerization, followed by the recruitment of pro-caspase-1 to the complex. Pro-caspase-1 then undergoes autolysis to produce active caspase-1 (19). Caspase-1, an IL-1-converting enzyme, then cleaves pro-IL-1β and pro-IL-18 into their mature forms, IL-1β and IL-18, respectively (1).

Caspase-1 associated inflammasomes are often termed canonical inflammasomes, while other caspases form noncanonical inflammasomes. These noncanonical inflammasomes are dependent on caspases-4/5 in humans and caspase-11 in mice and form in response to lipopolysaccharide (LPS) from bacteria (20–22). This review will focus on the caspase-1-dependent canonical inflammasomes.

In addition to cleaving IL-1β and IL-18, caspase-1 also cleaves GSDMD. GSDMD in turn forms pores in the plasma membrane, leading to cell death through pyroptosis and release of IL-1β and IL-18 (23). There is some debate as to whether GSDMD pore formation is a terminal event that necessarily leads to cell death and whether there are other mechanisms of IL-1β and IL-18 release. The nuances of these arguments are beyond the scope of this review.

## HERPESVIRUSES ARE COMMON HUMAN PATHOGENS

The human herpesviruses (HHV) are capable of causing a wide range of diseases, from asymptomatic infection to oncogenesis, retinitis, and lethal encephalitis. HHV virions consist of (i) an icosahedral capsid surrounding the dsDNA genome, (ii) a largely unstructured proteinaceous layer called the tegument that surrounds the capsid, and (iii) an outer lipid bilayer envelope studded with glycoproteins. The hallmark of HHVs is their ability to cause lifelong latent infections, and they are subdivided into three subfamilies known as alpha-, beta-, and gammaherpesvirinae (24).

Alphaherpesviruses consist of HSV-1 and HSV-2 (*Human alphaherpesvirus 2*, HHV-2), which replicate in cells and tissues of many mammalian species, and varicella-zoster virus (*Human alphaherpesvirus 3*, VZV/HHV-3), which can only replicate in cells of human or simian origin. All three viruses have broad cellular tropism, but become latent in ganglia along the entire neuraxis, from which they can reactivate to cause recurrent viral shedding or disease (24). HSV-1 and HSV-2 are quite prevalent, with more than half the adult population infected with one or both viruses (25–28). VZV is common worldwide, with seroprevalence rates of >90% in most populations (29). HSV-1- and HSV-2-related disease ranges from mild mucocutaneous lesions in the oral and genital mucosa to vision-threatening keratitis and life-threatening encephalitis. VZV is the etiologic agent of varicella (chickenpox) and herpes zoster (shingles) and can cause devastating disease in special populations, including immunocompromised hosts (30).

Betaherpesviruses include CMV, HHV-6A (*Human betaherpesvirus* 6A), HHV-6B (*Human betaherpesvirus 6B*), and HHV-7 (*Human betaherpesvirus 7*), which can establish latent infection in lymphocytes and other hematopoietic cells (31). In the United States, 40 to 60% of individuals are infected with CMV by adulthood, with seroprevalence approaching 100% in some parts of the world (32, 33). Of these, CMV is the most clinically relevant as it is a major cause of neonatal complications and morbidity in immunosuppressed populations (31, 34, 35).

The gammaherpesvirus subfamily includes Epstein-Barr virus (*Human gammaherpesvirus 4*, EBV/HHV-4) and Kaposi’s sarcoma-associated herpesvirus (*Human gammaherpesvirus 8*, KSHV/HHV-8). The prevalence of EBV is 70 to 95% in adults worldwide, with infection usually occurring during childhood. EBV persists mostly in memory B cells. Primary infection is often asymptomatic, but can lead to mononucleosis in adolescents and adults. EBV is also associated with a number of malignancies, including nasopharyngeal carcinoma (NPC) and Burkitt’s lymphoma (BL) (24, 30, 36). KSHV seroprevalence is high in sub-Saharan Africa (30% to 50%) and in the Mediterranean region. It is the etiological agent of the most common AIDS-related malignancy, Kaposi’s sarcoma (KS), as well as primary effusion lymphoma (PEL), multicentric Castleman disease (MCD), and KSHV inflammatory cytokine syndrome (KICS), all of which primarily occur in immunocompromised patients (37, 38).

## INFLAMMASOME ACTIVATION HAS VARIABLE IMPACT ON DISEASES CAUSED BY HERPESVIRUSES

Viral activation of the inflammasome is common, including by influenza (39), hepatitis C (HCV) (40, 41), HIV (42), and herpesviruses. Data regarding the role of inflammasome activation in the pathogenesis of alphaherpesviruses in humans are limited. However, evidence in murine HSV-1 and HSV-2 infection models supports a central role for inflammasome activation in both control and pathogenesis. IL-1β knockout (KO) mice are significantly more susceptible to lethal HSV-1 encephalitis than wild-type (WT) controls (43). This indicates that the IL-1β produced by monocytes/macrophages early during infection is critical for protection from overwhelming disease (44). Similarly, IL-18 is essential for NK cell activation (45) and protection from both lethal HSV-1 pneumonitis (46, 47) and HSV-2 genital disease (48, 49). Furthermore, IL-18 may help ameliorate eye lesions in herpes stromal keratitis (HSK) (50). In keeping with the hypothesis that the inflammasome plays a protective role in HSV disease, a study using a murine model of HSK found that NLRP3 KO mice had more severe HSK lesion development than WT mice (51).

However, several recent studies have found that inflammasome activation in the context of HSV-1/HSV-2 infection may be detrimental to the host. IL-1β can lead to HSV-1 reactivation in neurons (52). Furthermore, more virulent strains of HSV-1 cause more severe corneal pathology that is associated with increased IL-1β and IL-18 levels (53), and IL-18 contributes to HSV-2 pathology in a genital model of infection (54). Finally, HSV-1 infection of ASC KO and NLRP3 KO mice led to decreased inflammation and mortality compared to WT controls in a model of encephalitis (17). These findings are consistent with those of a study in humans which found that a ratio of high IL-1β in the cerebral spinal fluid to low IL-1 receptor antagonist (IL-1RA) in plasma was associated with a poor outcome in HSV-1 encephalitis (55). The differences between studies showing both protective and pathogenic roles for inflammasome activation during HSV-1 and HSV-2 infection may reflect the distinct effects of inflammasome cytokines at different disease sites, the opposite effects of moderate versus very high levels of inflammasome activation, or other unknown factors. Additionally, there can be non-inflammasome sources of IL-1β and IL-18, making it challenging to consistently draw direct links between inflammasome activation and pathological observations associated with IL-18 and IL-1β in herpes infections (7, 56, 57). Regardless, these data highlight the complex interactions between intracellular signaling pathways and immune response coordination, and underscore the need for additional research on the impact of inflammasome signaling in the context of HSV-1 and HSV-2 infection in both human and animal models.

Very little is known about the impact of inflammasome activation in VZV disease in humans, but an investigation with human skin xenografts in a SCID mouse model of VZV revealed that NLRP3 was induced in cells within VZV lesions, suggesting that VZV-induced inflammasome activation takes place *in vivo* (58). More recently, a study found that pharmacologically reducing endoplasmic reticulum stress in a rat model of VZV-associated post-herpetic neuralgia (PHN) led to decreased IL-1β and IL-18 release and improved PHN scores, suggesting that long term activation of the inflammasome is associated with more severe VZV-induced PHN (59). This, combined with the results for HSV-1 which demonstrate a detrimental effect of inflammasome activation (particularly in encephalitis), suggest a model where inflammasome activation in the periphery helps control viral replication, but inflammasome signaling in neuronal tissue leads to pathological inflammation that is harmful to the host. Additional studies will need to be done to refine this hypothetical model.

The impact of inflammasome activation during CMV infection is also somewhat unclear. A study of CMV viremia in CMV-seronegative (R-) kidney transplant recipients who received a kidney from a seropositive (D+) donor found that IL-18 increased along with other proinflammatory cytokines during viremia, suggesting that CMV activates inflammasomes during acute infection (60). More recently, IL-18 was shown to be associated with more severe CMV disease in solid organ transplant recipients regardless of donor and recipient serostatus (61), and certain polymorphisms in the IL-18 promoter are associated with an increased likelihood of CMV reactivation after stopping prophylaxis in kidney transplant recipients (62). These studies demonstrate that, at least in the immunocompromised population, CMV infection likely leads to inflammasome activation *in vivo*, associated with CMV-induced pathology, and that IL-18 regulation is related to CMV control.

The other betaherpesvirus that is often studied clinically is HHV-6. Though data on HHV-6 and inflammasome activation are lacking for the immunocompromised population, a small study did identify a potential link between HHV-6 copy number and IL-1β levels in children with febrile seizures. Previous studies showed that HHV-6 DNA is detectable in blood from a minority of patients that suffer febrile seizures (63) and that higher levels of IL-1β were found in the saliva of children with seizures, with HHV-6 copy number and IL-1β in saliva positively correlated (64).

Regarding inflammasome activation in EBV-related diseases, EBV-induced infectious mononucleosis leads to elevated IL-18 levels in plasma and substantial amounts of IL-18 in lymphoid tissues (65, 66). Similarly, IL-1β is elevated in the tonsils of children acutely infected with EBV (67). These early studies showed that acute EBV infection is associated with inflammasome-driven cytokines *in vivo*. In latent infection, high IL-18 is associated with regression of BL tumors in a murine model (68). Similarly, IL-18 receptor expression is upregulated in EBV-infected BL (69). In EBV-associated NPC, increased inflammasome gene expression was associated with improved survival, and IL-1β inhibited tumor growth in a murine model of the disease (70). Additionally, a recent study demonstrated that inflammasome activation in EBV positive tumor cells leads to lytic replication (71). Therefore, while these studies suggest that inflammasome activation in EBV latency-associated malignancies may be beneficial and lead to decreased tumor burden, it is unclear whether this is a direct effect of inflammasome activation or an indirect effect of lytic replication of the virus.

There is ample evidence of inflammasome activation in diseases caused by KSHV. Proinflammatory cytokines, such as IL-1β, IL-6, and TNF-α, are known to promote the pathogenesis of KSHV-associated diseases (72). IL-1β is elevated in patients with KS and promotes tumorigenesis when added to KS cells in culture. IL-1β also increases resistance to apoptosis, potentially promoting tumor survival (73, 74). MCD flares are also associated with an increase in IL-1β (75), and PEL cells constitutively produce IL-1β (76). Similarly, HIV-infected men with KSHV associated diseases have higher IL-18 and inflammasome activation in monocytes compared to healthy controls (77). Therefore, in contrast to EBV-associated malignancies, it appears that inflammasome activation promotes oncogenesis in KSHV-associated proliferative disorders.

## MULTIPLE INFLAMMASOMES ARE TRIGGERED IN RESPONSE TO HERPESVIRUSES

The importance of IL-1β and IL-18 in herpesvirus pathogenesis has long been known, but additional techniques for querying the inflammasome were required to demonstrate that HSV-1 directly activates the inflammasome. During early investigations of the AIM2 inflammasome, it was discovered that HSV-1 activates the inflammasome in macrophages without requiring this dsDNA sensor (78). Subsequently, it was found that a viral protein, VP22, specifically inhibits the AIM2 inflammasome during HSV-1 infection (79). Thus, other sensors have been proposed to function as inflammasome sensors for HSV-1 infection. While there is heterogeneity within the literature, NLRP3 has been consistently found as central to HSV-1 inflammasome activation. This has been demonstrated in keratinocytes (80), human foreskin fibroblasts (HFFs) (16), and macrophages (15). The mechanism by which HSV-1 activates NLRP3 is thought to be mediated through stimulator-of-interferon-genes (STING). STING recruits NLRP3 to the endoplasmic reticulum and attenuates NLRP3 K48- and K63-linked polyubiquitination, thereby promoting inflammasome activation (81). Whether other inflammasomes are activated in response to HSV-1 depends on the specific models of infection studied. The AIM2 inflammasome has been proposed to function in keratinocyte infection (80) and in some mouse models (82). Gamma-interferon-inducible protein 16 (IFI16) sensing of HSV-1 is thought to lead to inflammasome activation in HFFs (16). However, neither AIM2 nor IFI16 are required for inflammasome activation in macrophages (15). These and other inflammasome proteins, including NLRP12, are upregulated during HSV-1 infection in mice, but whether or not they are required for or directly involved in HSV-1-induced inflammasome activation remains unclear (53). Thus, while NLRP3 appears to be the primary inflammasome activated in HSV-1 infection, HSV-1 may activate other inflammasomes in a subset of cell types or tissues.

Apart from the known AIM2-inibitory function of VP22, it is not entirely clear if HSV-1 encodes other inflammasome inhibitory or regulatory elements. ICP0 is thought to attenuate NLRP3 and IFI16 induction in HFFs (16), and there is evidence that some replication-dependent factor inhibits NLRP3 inflammasome activation in macrophages (15). However, the direct impact of inflammasome activation on HSV-1 replication is unclear. As a result, how viral regulation of inflammasome activation affects the viral life cycle and pathogenesis is an open area of investigation.

Few studies have investigated the mechanism of VZV-induced inflammasome activation, but there are data suggesting that it activates the NLRP3 inflammasome in at least three different cell types: primary lung fibroblasts, a human melanoma cell line, and the monocyte/macrophage-like THP-1 cell line, all of which are permissive for VZV replication *in vitro* (58).

Although it was known that CMV strongly activates innate immune signaling by expressing proinflammatory cytokines and the secretion of IL-1β in multiple cell types (83–85), it was only recently demonstrated that CMV activation of the inflammasome is dependent on AIM2 and enhanced by STING (18, 86). Interestingly, IL-1β inhibits *in vitro* growth of CMV (87), and CMV immediate early 86-kDa protein (IE-86) inhibits IL-1β release from CMV-infected cells (18). This suggests that regulation of the inflammasome during CMV infection is critical for the viral life cycle and pathogenesis.

Studies on HHV-6 induction of inflammasome signaling *in vitro* are limited, but HHV-6 infection of peripheral blood mononuclear cells leads to IL-1β production (88). Both HHV-6A and HHV-6B infection of T cells in culture also leads to upregulation of the *IL-18* gene (89). Given these findings and what is known about CMV activation of the inflammasome, it is likely that HHV-6 activates the AIM2 inflammasome, but definitive studies are lacking.

EBV infection of both THP-1 cells and primary human monocytes leads to the release of IL-1β, suggesting that EBV is capable of activating inflammasomes in monocytes. EBV infection of these cells leads to upregulation of AIM2, and knockdown of AIM2 attenuates IL-1β release, indicating that EBV activates the AIM2 inflammasome in monocytes (90). However, in B cell infection, IFI16 interacts with the EBV genome and leads to ASC-dependent inflammasome activation (91–93). While not implicated in EBV-induced inflammasome activation, NLRP3 signaling does lead to EBV reactivation from latency in cell culture models (71). Therefore, it is possible that multiple inflammasomes play an important role in EBV replication and cell biology.

In contrast, multiple studies have indicated that KSHV activates the inflammasome in both epithelial cells and B cells through an IFI16-dependent mechanism (76, 93, 94). KSHV also activates the inflammasome in latency-associated malignancies, including KS and PEL (76). The activation of inflammasomes by KSHV is facilitated and regulated by BRCA1 (93). KSHV also has evolved multiple mechanisms to regulate inflammasome activation. The KSHV protein Orf63 is known to block NLRP1-dependent inflammasome activation, and possibly NLRP3 as well, as a way to promote efficient reactivation from latency (95). The KSHV polyadenylated nuclear RNA (PAN RNA) can modulate the innate immune response by decreasing pro-IL-18 mRNA (96). KSHV latency-associated nuclear antigen (LANA) also has a known caspase-1 cleavage site and functions to blunt IL-1β production (97). Furthermore, there is evidence that release of IFI16 and IL-1β in exosomes is another potential mechanism by which KSHV subverts the host innate response, though the implications of this finding are not yet known (76). Taken together, these studies demonstrate that IFI16 is at the center of KSHV inflammasome biology, yet there is a complex interaction between KSHV and the inflammasome response in viral persistence and pathogenesis which is not fully defined. A summary of various inflammasome activation impacts on herpes virus-associated diseases is provided in Table 1.

**TABLE 1:** Impact of inflammasome activation on human herpesvirus-associated diseases

Virus	Inflammasome activation		Sensor	Clinical disease	Impact on disease outcome	References
	*In vitro*	*In vivo*				
HSV-1 (HHV-1) and HSV-2 (HHV-2)	Yes	Yes	NLRP3, AIM2, IFI16	Encephalitis	IL-1β KO worsens disease in mice	15–17, 43–50, 52–55, 80–82
					↑CSF IL-1β and periphery IL-1RA associated with worse disease in humans	
					ASC KO and NLRP3 KO decrease inflammation and death in mice	
				Pneumonitis	↑IL-18 improves disease outcomes	
				Herpes stromal keratitis (HSK)	↑IL-18 ameliorates disease	
				HSV-1 reactivation	IL-1β can increase reactivation in neurons	
				Genital disease	Unclear: IL-18 can be protective but also contributes to pathology	
VZV (HHV-3)	Yes	Yes (in murine models)	NLRP3	Post-herpetic neuralgia (PHN)	↓IL-1β and ↓IL-18 decrease disease	58, 59
CMV (HHV-5)	Yes	Yes	AIM2	Cytomegalovirus (CMV) in transplant patients	↑IL-18 associated with severe disease	18, 60–62, 83–86
HHV-6	Unknown	Yes	AIM2 (presumed)	Febrile seizures	↑IL-1β in children with febrile seizures	63, 64, 88, 89
EBV (HHV-4)	Yes	Yes	AIM2, IFI16	Burkitt lymphoma (BL) tumors in mice	↑IL-18 can lead to tumor regression	65–71, 90–93
				Nasopharyngeal carcinoma (NPC) tumor growth in mice	↑IL-1β inhibits tumor growth	
KHSV (HHV-8)	Yes	Yes	IFI16	Kaposi’s sarcoma (KS)	↑IL-1β can promote tumorigenesis	72–76, 93, 94
				Multicentric Castleman’s Disease (MCD)	↑IL-1β associated with MCD flares	
				Primary effusion lymphoma (PEL)	IL-1β constitutively expressed by PEL cells	

## CONCLUSIONS

The available evidence indicates that the interplay between herpesviruses and inflammasome signaling is central to both the viral life cycle and the pathogenesis of herpesvirus-related diseases. However, it is also clear that both the mechanism of inflammasome activation and the impact of that activation on the host are unique to each herpesvirus and may be unique to the specific cell or tissue that is infected. Therefore, studies of one herpesvirus cannot be generalized to other members of the herpesvirus family and should be interpreted with caution regarding other models of infection for that same virus. Additional investigations into the impact of inflammasome activation on diseases caused by herpesviruses and how herpesviruses activate and regulate inflammasomes are critical. This is particularly true as inflammasome modulators make their way through clinical trials (98). Whether these therapeutics can be used to improve herpesvirus-related diseases or whether they may exacerbate these pathologies is an open question. Given the high prevalence of HHV infections, the scientific community should investigate the effects of inflammasome modulators on herpesvirus-associated disease prior to their wide-spread use.
